# Structure and substrate promiscuity of *Campylobacter jejuni* periplasmic nitrate reductase (Nap) and phylogenetic analysis of Nap homologs

**DOI:** 10.1016/j.jbc.2025.110928

**Published:** 2025-11-15

**Authors:** Nitai C. Giri, Trung Thach, KanagaVijayan Dhanabalan, Mintare Cesiunaite, Manohar Radhakrishnan, Lahiru Wedasingha, Nicholas Manicke, Michael Wells, Maciej Szaleniec, Ramaswamy Subramanian, Partha Basu

**Affiliations:** 1 Department of Chemistry and Chemical Biology, Indiana University, Indianapolis, Indiana, USA; 2 Department of Biological Sciences, Purdue University, West Lafayette, Indiana, USA; 3 School of Biological Sciences, Louisiana Tech University, Ruston, Louisiana, USA; 4 Jerzy Haber Institute of Catalysis and Surface Chemistry, Polish Academy of Sciences, Kraków, Poland

**Keywords:** cryo-EM, molybdenum enzymes, periplasmic nitrate reductase, structure-function

## Abstract

Periplasmic nitrate reductase NapA is a member of the DMSO reductase (DMSOR) superfamily, which catalyzes the reduction of nitrate to nitrite. *Campylobacter jejuni* NapA (*Cj*NapA) is notably larger compared to other structurally characterized NapA. Herein, we present the cryo-EM structure of *Cj*NapA, the first of its kind from any ε-proteobacteria, revealing three lysine-rich insertions that could affect the substrate channel, potentially enhancing the affinity towards nitrate and other anionic substrates. Here, we report that wild-type *Cj*NapA and NapA-C176D variants can reduce chlorate, perchlorate, and nitrate. However, the perchlorate and chlorate reductions by the *Cj*NapA C176D variant are considerably slower, even though the perchlorate reductase has an Asp coordination to Mo. Molecular Dynamics (MD) simulations were performed to investigate the impact of the C176D mutation on substrate affinity and protein flexibility. Structural and kinetic comparisons with perchlorate reductase support evolutionary tuning for a desired function. Finally, structural comparisons with other structurally characterized NapAs also suggest the role of proximal pterin in *Cj*NapA in electron transfer to the Mo center.

The dimethylsulfoxide reductase (DMSOR) superfamily is the most diverse among molybdenum enzyme families, comprising over 40 enzymes that catalyze essential life-sustaining reactions and mediate key processes in the global carbon, nitrogen, and sulfur biogeochemical cycles (1, 2, 3, 4). These reactions occur at the molybdenum center, where the Mo atom is coordinated by two pyranopterin (PDT) cofactors, forming the molybdenum cofactor (Moco) (5, 6). Most DMSOR family members also contain a [4Fe4S] cluster, leading to the designation as complex iron-sulfur molybdoenzymes (CISMs) (7). Crystal structures of several DMSOR enzymes have revealed critical structural differences, such as i) protein-derived amino acid ligands of the Mo atom (*e.g.*, Cys in periplasmic nitrate reductase, NapA; Sec in formate dehydrogenase, Fdh; Asp in perchlorate reductase, Pcr; Ser in DMSO reductase), ii) the conformation of the pyran ring of the pyraopterin cofactor (*e.g.*, NapA contains two tricyclic pyranopterin rings, while Pcr has one bicyclic and one tricyclic ring), and iii) residues in the second coordination sphere involved in hydrogen bonding (8, 9).

Members of the DMSOR superfamily exhibit substrate promiscuity (10), although non-native substrates generally show lower catalytic turnover rates (k_cat_) and efficiencies (k_cat_/K_m_) than native substrates, reflecting the evolutionary optimization for natural substrates. For example, the k_cat_ of *Cj*NapA for the non-native substrate TMAO is ∼0.1 s^–1^, with a catalytic efficiency of 5.7 × 10^2^ M^–1^s^–1^, compared to the k_cat_ of *Escherichia coli* TMAO reductase (TMAOR) for TMAO, which is 2500 s^–1^ with a catalytic efficiency of 1.2 × 10^7^ M^–1^s^–1^ (11, 12, 13). However, certain non-native substrates are turned over more efficiently in select cases. For instance, *Aspergillus oryzae* Pcr reduces nitrate with a k_cat_ of 0.85 s^−1^ (9), higher than its native substrate perchlorate (0.45 s^−1^), though its catalytic efficiency for nitrate (3.7 × 10^4^ M^−1^s^−1^) is much lower than that of *Cj*NapA (1.7 × 10^6^ M^−1^s^−1^). Here, we show that *Cj*NapA can reduce chlorate and perchlorate, in addition to nitrate . Additionally, ESI-MS analysis demonstrated that *Cj*NapA reduces perchlorate to chlorate and chlorate to chlorite. The high K_m_ values for chlorate and perchlorate imply that *Cj*NapA minimizes chlorite toxicity in the absence of chlorite dismutase.

The evolutionary origin of nitrate reductase has been explored previously (14, 15). These reports suggest that heterodimeric NapA (paired with NapB) evolved from assimilatory nitrate reductase (NasA) *via* a monomeric NapA intermediate (lacking *napB* in the operon). Monomeric NapA enzymes, such as *Dechloromonas desulfuricans* NapA, are smaller than heterodimeric NapAs, like those of *Cupriavidus necator* or *E. coli*, that have a couple of polypeptide insertions (15). The structural analysis reported here revealed six surface inserts in *Cj*NapA, three of which are part of the substrate channel and are enriched with positively charged residues, such as Lys. We suggest that these features contribute to the high affinity of *Cj*NapA for nitrate (13).

## Results

### Overall structure of *Cj*NapA

**Figure 1:**
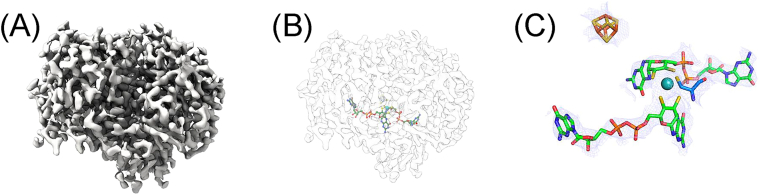
**The cryo-EM structure of CjNapA.** (*A)* the representative cryo-EM map showing the overall structure, (*B*) relative position of Moco from the surface, and (*C*) the prosthetic groups – Moco (*Mo is sphere*) and [4Fe4S] cluster (*cube*).

The overall structure (Fig. 1*A*) of *Cj*NapA is ellipsoidal, with the Mo-center situated approximately 15 Å below the NapA surface (Fig. 1*B*). In addition to Moco, NapA has a [4Fe4S] cluster, which is located closer to the surface of the protein and is coordinated by four cysteine residues: Cys42-X_2_-Cys45-X_3_-Cys49-X_27_-Cys77. These cysteine residues are conserved across both Gram-positive and Gram-negative bacteria. The distance between the Mo center and the nearest Fe atom of the [4Fe4S] cluster is 12.2 Å.

The Mo center is coordinated by five sulfur atoms (Fig. 1*C*). One of these sulfurs is provided by Cys176 of NapA, while the other four sulfur atoms are provided by two ene-dithiolene moieties of the pyranopterin cofactors. These Mo-S distances are consistent with those observed in nitrate-treated *Cj*NapA using EXAFS (16). Although the cryo-EM structure cannot resolve the identity of the terminal ligand, EXAFS analysis revealed that the terminal group is an oxo group in the Mo(VI) state, with a Mo=O distance of 1.73 Å (16). The PDT cofactors are spread along the interior of NapA, with a maximum distance of 31.7 Å between the C=O of two guanines and are stabilized by H-bonding interactions with several residues in the enzyme. Similar to other structures of NapA (17, 18, 19, 20), the pterin units in *Cj*NapA are tricyclic.

### Perchlorate and chlorate reductase activity

**Figure 2:**
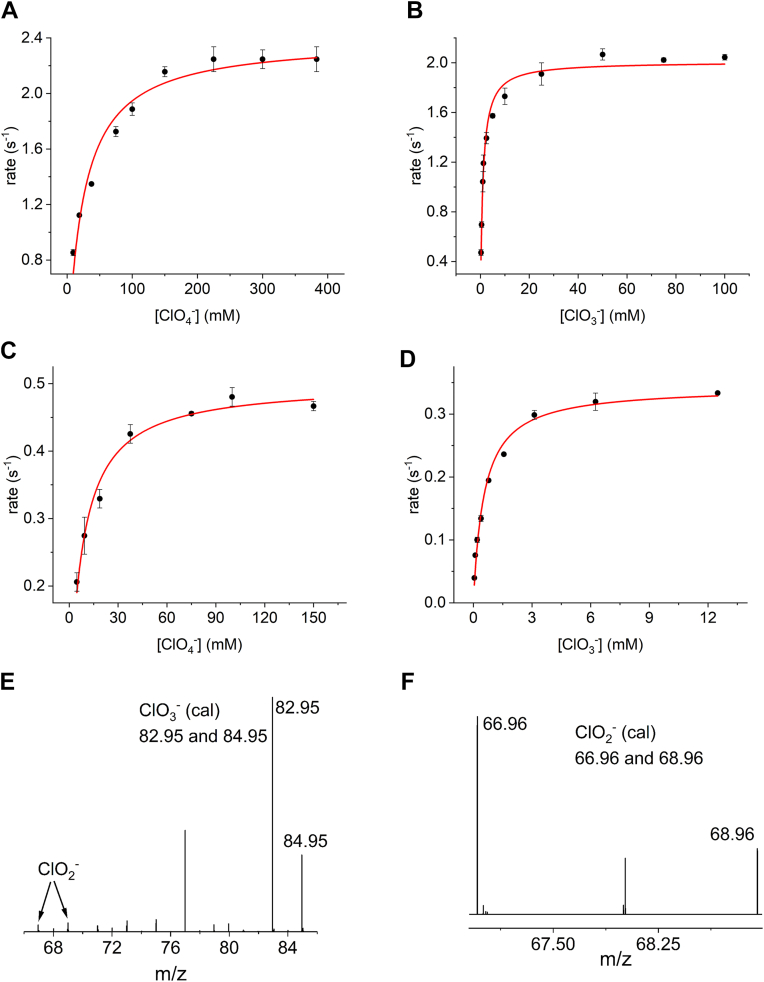
*Top*: Steady-state activity measurements (Michaelis-Menten model) of WT *Cj*NapA with perchlorate (*A*) and chlorate (*B*) as substrates. *Middle*: steady-state activity measurements of *Cj*NapA-C176D variant with perchlorate (*C*) and chlorate (*D*) as substrate; *Bottom*: ESI-MS analysis of the product of WT NapA catalyzed perchlorate (*E*) and chlorate (*F*) reduction shown in *top panels*.

Steady-state activity measurements were performed using perchlorate and chlorate as substrates. For perchlorate, a k_cat_ value of 2.5 s^−1^ was determined for the WT NapA (Fig. 2*A*), whereas the NapA-C176D variant (Fig. 2*C*) showed a k_cat_ of 0.51 s^−1^. Both values are lower than that for nitrate reduction catalyzed by WT NapA (5.91 s^−1^) (Table 1) (13). The K_m_ values of WT NapA and the NapA-C176D variant were determined to be 23 mM and 7.7 mM, respectively. These values are significantly higher than those for nitrate (3.4 μM) (13). The analysis of ESI-MS (Fig. 2*E*) indicates that the product of WT NapA-catalyzed perchlorate reduction is mostly chlorate, with a small amount (∼3%) of chlorite.

**Table 1:** Steady-state kinetic parameters

	*C. jejuni* WT NapA			*C. jejuni NapA-C176D*			*A. oryzae* PcrAB (9)		
Substrate	k_cat_ (s^−1^)	K_m_ (μM)	k_cat_/K_m_ (M^−1^s^−1^)	k_cat_ (s^−1^)	K_m_ (μM)	k_cat_/K_m_ (M^−1^s^−1^)	k_cat_ (s^−1^)	K_m_ (μM)	k_cat_/K_m_ (M^−1^s^−1^)
Nitrate	5.9 ± 0.18^a^	3.4 ± 0.44^a^	1.7 × 10^6^	0.34 ± 0.01^b^	462 ± 66^b^	7.4 × 10^2^	0.85	23	3.7 × 10^4^
Chlorate	2.13 ± 0.04	968 ± 93	2.2 × 10^3^	0.35 ± 0.01	549 ± 65	6.4 × 10^2^	0.45	7.4	6.1 × 10^4^
Perchlorate	2.52 ± 0.07	22907 ± 3132	1 × 10^2^	0.51 ± 0.01	7678 ± 855	6.6 × 10^1^	0.45	6.0	7.5 × 10^4^

a The values were taken from reference (13).

b The values were taken from reference (11).

Steady-state activity measurements performed with chlorate as a substrate indicate k_cat_ values of 2.1 s^−1^ and 0.35 s^−1^ for WT NapA (Fig. 2*B*) and NapA C176D variant (Fig. 2*D*), respectively, both of which are also lower than that for nitrate reduction catalyzed by WT NapA (5.91 s^−1^) (Table 1) (13), The K_m_ values of WT (∼1 mM) and C176D (∼0.5 mM) NapA for chlorate are lower than those for perchlorate. ESI-MS analysis revealed that the product of the chlorate reduction catalyzed by WT NapA is chlorite (Fig. 2*F*).

### Inhibition of WT NapA by perchlorate

**Figure 3:**
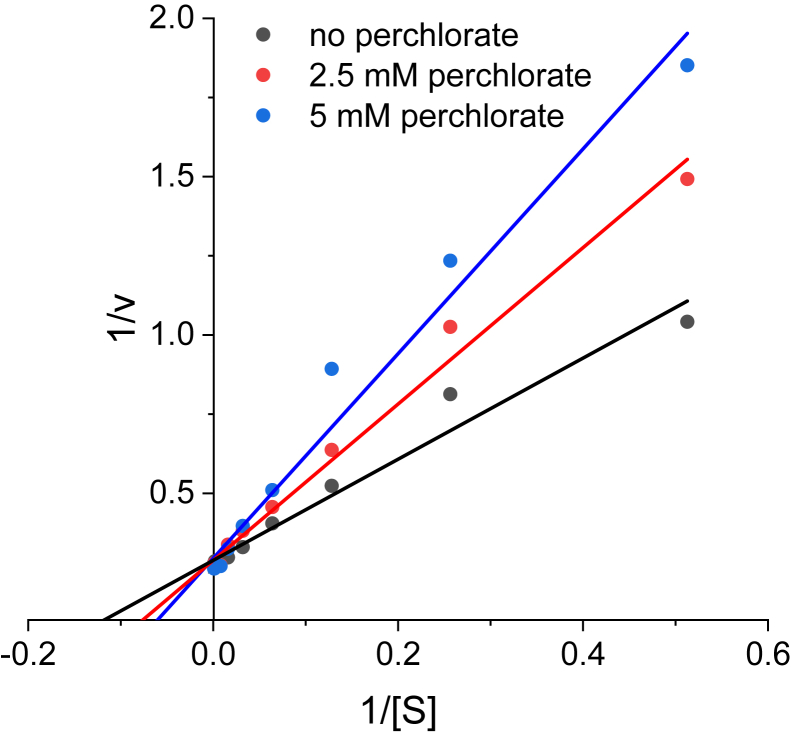
**Double-reciprocal plot showing competitive Inhibition of NapA by perchlorate.** The enzyme concentration was 67.5 nM in 50 mM HEPES at pH 7.

We have also investigated the inhibitory effect of perchlorate on nitrate reduction. The double reciprocal plot of 1/v vs 1/[S]_nitrate_ at different perchlorate concentrations is shown in Figure 3. The results show an increase in K_m_ with increasing concentrations of perchlorate. However, V_max_ does not change significantly with changes in perchlorate concentration. Overall, these results indicate competitive inhibition of NapA by perchlorate with an apparent K_i_ of 4.7 ± 0.3 mM.

### Phylogenetic analysis

**Figure 4:**
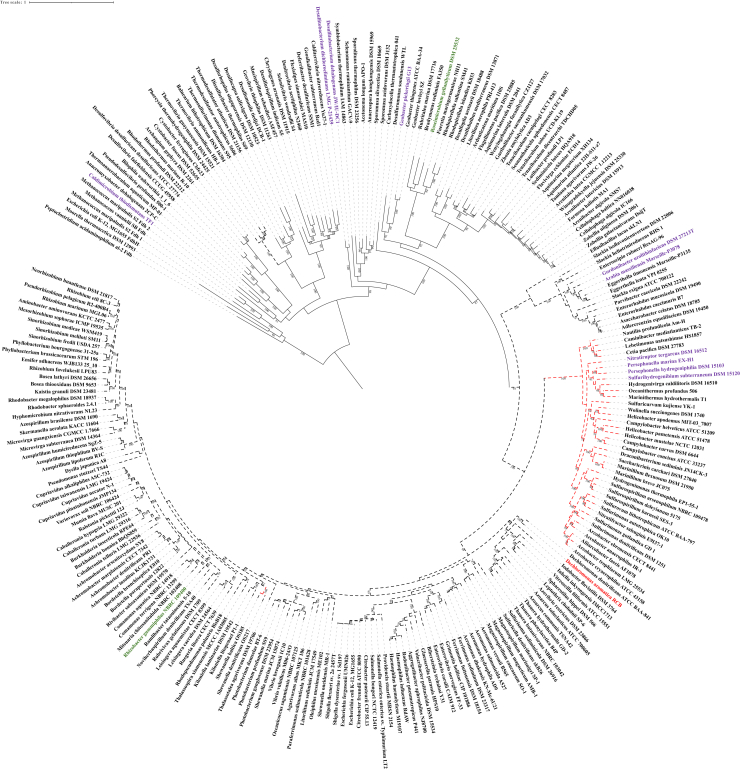
**Maximum likelihood phylogeny of the NapA family in the DMSOR superfamily.***Solid black* branches indicate that the NapA sequence in the organism’s genome is ∼750 to 800 amino acids long. *Dotted black* branches indicate that the NapA sequence in the organism’s genome is ∼850 to 875 amino acids long. *Dotted red* branches indicate that the NapA sequence in the organism’s genome is ∼925 to 975 amino acids long. *Purple* branch labels indicate that an organism’s genome contains a perchlorate or chlorate reductase with no known chlorite dismutase homolog. *Red* branch labels indicate that an organism’s genome contains both a perchlorate or chlorate reductase and chlorite dismutase. *Green* branch labels indicate that an organism’s genome contains no perchlorate or chlorate reductase but does harbor a *Nitrospira*-type chlorite dismutase.

The phylogenetic analysis of NapA reveals that the differences in primary structure length correspond to evolutionarily meaningful clades (Fig. 4). Smaller NapA homologs, approximately 750 amino acids (mol wt. ∼83 kDa), represent the ancestral state of the NapA family. Longer sequence lengths evolved three times during the diversification of the NapA family. Sequence inserts of approximately 100 amino acids evolved twice. The first sequence insert appeared in the *Desulfurobacteriaceae* family of the *Aquificota* phylum. The second sequence insert encompasses a much broader range of radiation from the NapA family within the *Actinobacteriota* phylum, as well as α-, β-, and γ-proteobacterial NapA. Within this broader radiation of NapA sequences, NapAs of approximately 925 to 975 amino acids (∼105 kDa) evolved in radiation that comprised NapA from the *Campylobacterota* and *Thermus/Deinococcus* lineages, as well as the *Hydrogenothermaceae* family of the *Aquificota* phylum.

### MD simulations

MD simulations of WT and *Cj*NapA C176D variant were conducted to understand (i) the relative affinity of the enzyme toward different substrates, (ii) the influence of C176D mutation on the substrate affinity and protein flexibility, and (iii) to identify sites on the protein surface that attract ions or are involved in the substrate binding process. We also wanted to examine the path through which substrates diffuse into the active site and how the C176D mutation affects the transport process.

First, the behavior of NapA in the presence of 200 mM substrate ions was studied, during which we monitored the distance between the Mo atom and the N or Cl atom of substrate ions. Surprisingly, none of the ions was able to reach the van der Waals (vdW) distance of Moco, which could have exchanged the coordinated water and enabled binding of the substrate. Each of the ions only penetrated the pre-binding site at the entrance of the substrate channel (see Fig. S18). The shortest minimal distances were observed for chlorate, followed by perchlorate and nitrate (11.7, 12.7, and 14.6 Å, respectively, in Figs. S7, S9, and S5). However, an analysis of the radial distribution functions (RDFs) calculated for the Mo-N/Cl distance (Figs. S6*C*, S8*C*, and S10*C*) indicates the highest concentration of ClO_4_^-^ ions in the channel leading to the active site. The C176D mutation increased the likelihood of active site penetration, significantly reducing the minimal observed distances (9.3, 11.2, and 11.5 Å, respectively). The mutation especially increases the concentration of chlorate and perchlorate ions in the pre-binding site, approximately 12 Å away from the Mo atom (Figs. S6–S10). Further, for the NapA model with a pentacoordinate Mo-center in WT NapA (Figs. S7, S9, and S10), the shortest distances to Mo were observed for nitrate, followed by perchlorate and chlorate (10.8, 11,5, and 12.7 Å, respectively). At the same time, the NapA C176D variant did not show positive changes (11.5, 11.2, and 12.2 Å for NO_3_^-^, ClO_3_^-^, and ClO_4_^-^, respectively). The analysis of RDF corroborates the beneficial influence of the shift to a pentacoordinate Mo-center for penetration of the active site. This is especially prominent in the case of nitrate in the WT NapA, whereas the effect is moderate for the NapA C176D variant (Figs. S6–S10). These findings suggest that a shift in Moco coordination from hexacoordinate to pentacoordinate, triggered by breaking the weak Mo-OH_2_ bond, is most likely a critical step for transferring the native substrate from the pre-binding site to the actual binding site. Additionally, the NapA C176D mutation has a positive influence on chlorate and perchlorate binding and a mixed effect on nitrate binding.

The analysis of the root-mean-square-fluctuation (RMSF) of the main chain indicates that the C176D mutation has a globally negative influence on the flexibility of the main chain of the enzyme, while the change from the hexacoordinate to pentacoordinate cofactor increases the fluctuation of the main chain, and the positive increase is especially visible at main chain turns and loops, especially in the region over the entrance to the substrate channel (Fig. S11). This may indicate that the strain introduced by the shift in the metal coordination is propagated through the cofactor to the whole protein, which adjusts its global fold to accommodate the Moco better.

To further investigate the enzyme affinity, we modelled the behavior of enzyme-substrate complexes starting from the position of the nitrate obtained in the QM:MM study of the mechanism.

For the NapA with hexacoordinate Mo-center (Figs. S12, S14, S16), the median distance from the Mo atom was the lowest for perchlorate (6.2 Å), followed by nitrate and chlorate (8.0 and 8.2 Å, respectively) but with significantly broader distribution for the chlorate compared to nitrate (4.8–12.3 Å vs 5–9.2 Å). Both chlorate and perchlorate ions had longer residence times in direct contact with the Moco, while none of the ions were able to diffuse from the active site of the channel into the solvent during the simulation time; instead, they were retained in the pre-binding site (*e.g.*, Fig. S18). The change in conformation around the Mo-center opens up the enzyme for substrate dissociation, and the distance distributions shift from the direct vicinity of Moco toward their prebinding site (Figs. S13, S15, S17). Interestingly, the C176D mutation has a positive binding effect on chlorate and perchlorate, and an adverse binding effect on NO_3_^-^ for the hexacoordinate model; combined with the pentacoordinate binding mode results in a quick release of the substrate into the bulk solvent. This indicates that the NapA C176D variant would indeed exhibit a decrease in substrate selectivity of the enzyme, facilitating transfer of ions between the active site and bulk solvent.

## Discussion

### Structural comparison with other NapAs

**Figure 5:**
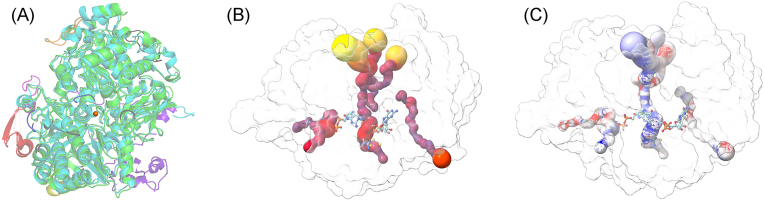
*A*, alignment of *Cj*NapA with CnNapAB (PDB ID: 3ml1), where *Cj*NapA is in *green*, *Cn*NapA in cyan and *Cn*NapB in *purple blue*. The extra loops in *Cj*NapA are shown in different colors. Three of these inserts (*blue*, *magenta* and *orange*) are part of the substrate channel. Mo is in *sphere*. *B*, the radius of the substrate channel (*dark red* <1 Å, red 2 Å and *yellow* >2 Å) and *C*: electrostatic potential analysis of the substrate channel (*Blue*: positive, *white*: neutral and *red*: negative).

The structure of NapA reported here is the first structure from the ε-Proteobacteria phylum (now known as the *Campylobacterota*). The alignments of the *Cj*NapA structure with available structures of NapA indicate that *Cj*NapA is more similar to heterodimeric NapA structures, as evidenced by the RMSD. The RMSDs are 0.87 Å with *Campylobacter sphaeroides* NapA, 0.84 Å with *E. coli* NapA, and 0.85 Å with *C. necator* NapA. In contrast, the monomeric *D. desulfuricans* NapA has an RMSD of 1.33 Å. *Cj*NapA has an additional ∼100 amino acid residues relative to NapA from other organisms. These additional amino acid residues can be viewed as polypeptide insertions. Alignment of the structures of NapA from *C. necator* and *Campylobacter jejuni* indicated that six insertions are located on the enzyme surface (Fig. 5*A*). Three of these inserts (blue, magenta, and orange) are located on the enzyme surface that is part of the substrate channel. Sequence analyses indicate that these inserts are rich in positively charged residues like Lys (blue: DFNKMLTNGDLIAPKDEKEHSI, magenta: NPNHPKFKESEKDTV, and orange: EQKVDNKLTLPS in Fig. 5*A*). These positively charged inserts can act as docking sites for negatively charged nitrates, resulting in a high affinity of nitrates for *Cj*NapA. This hypothesis was tested by MD simulation and the analysis of the radial distribution of NO_3_^-^ ions around positively charged Lys residues in a model solvent containing 0.2 M NaNO_3_. In almost all cases, ions formed the first coordination sphere around each Lys residue at 3.4 Å, resulting in 3- to 16-fold higher density of nitrate ions compared to bulk solvent (Fig. S19).

### Conformation of PDT moieties and their possible role in catalysis

**Figure 6:**
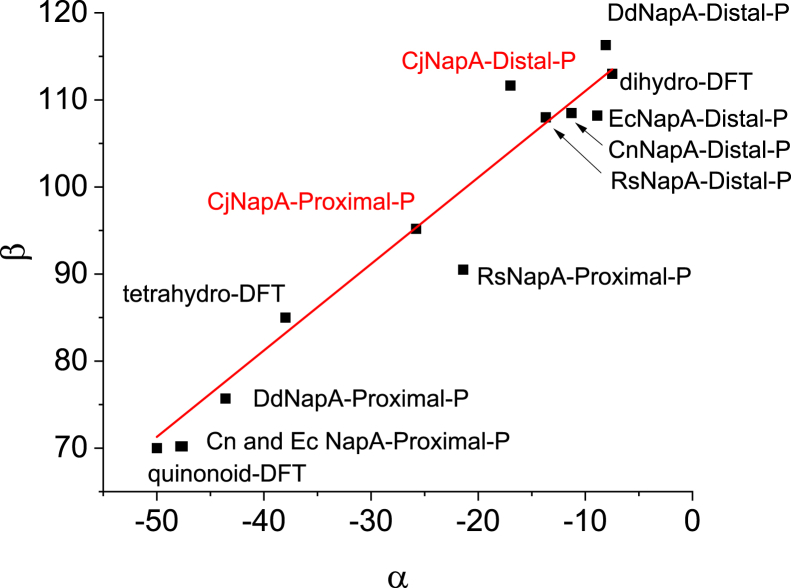
The conformations of proximal and distal pterins of *Cj*NapA and other NapAs from crystal structures and the calculated dihydro- and tetrahydro-pterins.

*Cj*NapA, like all other members of the DMSO family, has two PDT moieties attached to the Mo center. These two PDTs have been assigned as proximal and distal based on their positioning with respect to the [4Fe4S] cluster. The role of pterins in the catalysis of molybdenum enzymes remains an important topics of discussion (8, 21, 22, 23, 24, 25). These complex molecules comes in different flavors in different enzymes and even the same pterin adopt different conformations in enzymes containing two pterins (26). Previous analysis of pyranopterins in more than 100 structures of mononuclear molybdenum and tungsten enzymes indicated that the conformation of the pyranopterins can be described by two dihedral angles α and β (27). The plot of β vs α gives information about the planar or distorted nature of the pyranopterins, which can be related to the oxidation state of the pyranopterins (*e.g.*, dihydro, tetrahydro, *etc.*) (8, 26). This analysis indicated that the two pterins of the DMSOR family are different. This conclusion is supported by resonance Raman studies on *C. sphaeroides* DMSOR and *E. coli* biotin sulfoxide reductase (28, 29). The analysis of pyranopterins in *Cj*NapA indicates that the proximal pterin is more distorted than the distal pterin (Fig. 6), which is a common feature of the DMSOR family. It is worth noting that the calculated value of dihydropterin falls near the distal pterin in all the available NapA structures, including *Cj*NapA (Fig. 6). Thus, this analysis suggests that the distal pterin of *C. jejuni* NapA and other NapAs is consistent with a dihydro form. However, the dihedral values for the proximal pterin span a broad range. For example, the values for the proximal pterin in *Cj*NapA are closer to those of *Rs*NapA, while the values for *Cn*NapA, *Ec*NapA, and *Dd*NapA are close to each other. In this context, it is worth mentioning that the Mo(VI/V) potential is significantly lower in *Ec*NapA (−100–100 mV (20)) than that in *Rs*NapA (>300 mV (19)). Pushie *et al.* noted that correlating the redox states of the pyranopterin with protein crystal structures has inherent limitations due to structural resolution constraints (30). Moreover, factors such as the influence of the metal center and the surrounding protein environment, including hydrogen bonding interactions, may contribute to structural distortions. Therefore, caution should be exercised before drawing definitive conclusions. Nonetheless, the preceding discussion provides a valuable foundation, and future studies should aim to address these aspects to achieve a more comprehensive and precise understanding of pyranopterin redox chemistry.

It is generally considered that the proximal pterin is involved in electron transfer, and the distal pterin is thought to modulate the redox potential (31, 32). The distinct clustering of the distal pterin in the above plot and the scattered nature of the proximal pterin raise a question whether proximal pterin can also modulate the redox potential. Furthermore, it has been suggested that the pterin involved in electron transfer has conformations similar to the tetrahydro state (27). For example, the pyranopterin in xanthine dehydrogenase resembles the proximal pterin of the DMSOR family (27). It has been implicated in electron transfer to the Mo-center from the FAD *via* [2Fe-2S] clusters (33). The values for the proximal pterin in *Cj*NapA are closer to the calculated value of tetrahydropterin (Fig. 6), indicating that it might be involved in electron transfer to the Mo-center. Further support for this suggestion comes from the conserved Lys79 (Supporting Information, Fig. S4), which sits between the proximal pterin and the [4Fe4S] cluster, and the loss of nitrate reductase activity when this Lys is replaced by other residues such as Ala, Met, *etc.* (32, 34, 35) A similar situation has also been found in formate dehydrogenases (36, 37).

### Substrate channel in NapA

The deeply buried Mo center in NapA is connected to the bulk solvent *via* a substrate channel. This funnel-shaped substrate channel with a larger radius (yellow) narrows toward the active site (Fig. 5*B*). The nitrate-soaked structure of *D. desulfuricans* NapA shows the presence of nitrate in this channel (PDB ID: 2JIQ). Also, the structure of the perchlorate-bound *Dd* NapA shows the presence of perchlorate in this channel (PDB ID: 2V45). Nitrate and perchlorate are not bound to the Mo center in these structures. However, the closest nitrate and perchlorate occupy a similar position (∼10 Å away from the Mo center) in these structures. This is consistent with the MD simulations reported here, which suggest similar pre-binding sites in NapA at approximately 8 to 9 Å away from the Mo center (Fig. S17). It was proposed that perchlorate inhibition would occur by blocking the entry of the substrate to the catalytic site (38). The competitive inhibition by perchlorate observed here is consistent with this proposal, particularly in that perchlorate tends to penetrate deeper into the channel compared to NO_3_^-^, while exhibiting a similar range of Gibbs free energy of binding (ΔG_b_ of −27 ± 1.5 kcal mol^–1^ and –19.9 ± 1.7 for NO_3_^-^ and ClO_4_^-^, respectively, Fig. S20). Here, we also show that NapA can accept perchlorate and chlorate as substrates (although not as well as nitrate), as discussed below. The substrate channel in *Cj*NapA is defined by various conserved polar residues, such as Arg174, Asp191, Glu192, Arg463, Arg471, and His715. Electrostatic potential analysis of the substrate channel in *Cj*NapA (Fig. 5*C*) shows the prevalence of positively charged amino acid residues. Thus, a combination of positively charged amino acid residues and positively charged surface residues could lead to a higher affinity for negatively charged nitrate.

### Structural comparison with perchlorate reductase

**Figure 7:**
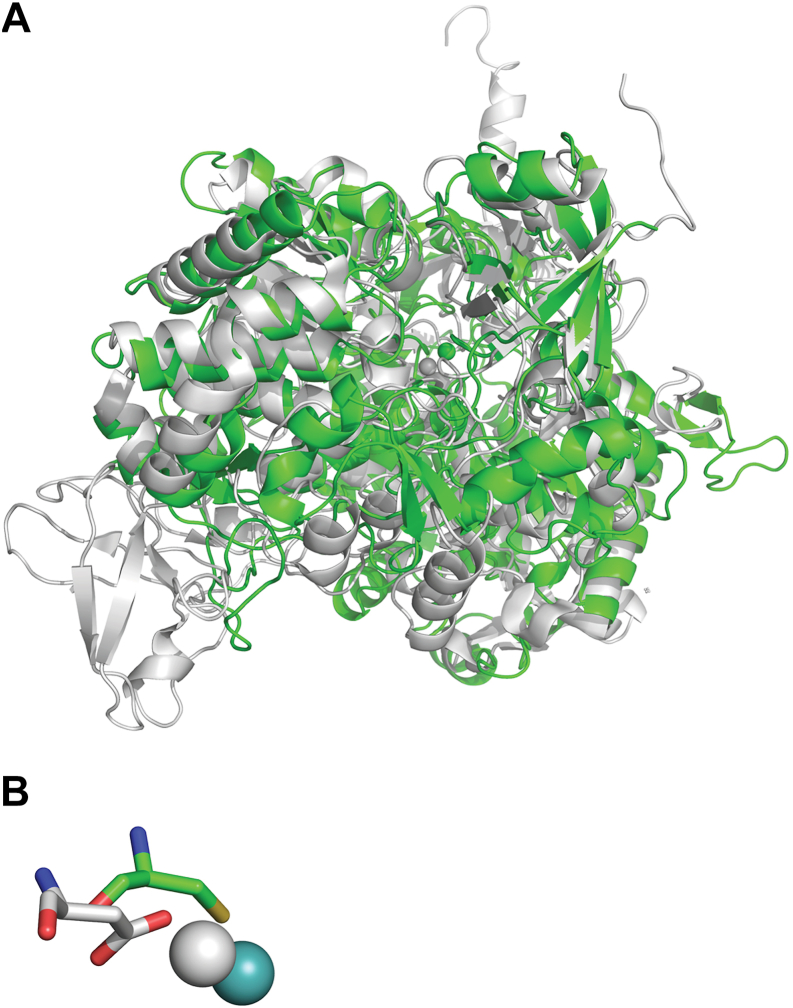
*A*, alignment of *Cj* NapA (*green*) structure with *Ao* PcrA structure (PDB ID: 5CHC, *gray*). *B*, conserved residues near the Mo center (*sphere*) in NapA (*cyan*) and PcrA (*gray*).

The protein-derived amino acid ligands in *Cj*NapA and *Ao*PrcA are Cys and Asp, respectively. Also, the structural similarity between *Cj*NapA and *Ao*PcrA is lower (RMSD = 6 Å) than that compared to the high similarity with the structures of other NapAs. This low structural similarity is consistent with the low sequence identity between *Cj*NapA and *Ao*PcrA (23.8%). In the SeO_3_^2-^ bound crystal structure of reduced perchlorate reductase from *A. oryzae*, one O-atom of SeO_3_^2-^ is bound to the Mo-center (Mo-O distance 2.2 Å) (9). However, in the nitrate-soaked structure of *D. desulfuricans* NapA, presumably in the oxidized state of Mo, no nitrate is bound to the Mo-center (38). These results imply that the substrate binds only to the reduced enzyme, which contains the Mo(IV) state, which is consistent with our biochemical results (39). However, upon reduction, the site may undergo a structural change allowing substrate binding. Interestingly, the Mo-binding Asp198 in *Ao*PcrA is in a similar position to Cys176, the Mo-binding residue in *Cj*NapA (Fig. 7). However, the nature of the residues bordering the substrate channel in *A. oryzae* PcrA (Pro, Phe, Tyr, Gln, Gly, Trp, Asn, *etc.*) is different from those of proteobacterial NapA (Arg, Asp, Glu, His, *etc.*, mentioned above), including *Cj*NapA. The aromatic residues in the substrate channel in PcrA have been associated with its unique biophysical parameters (*e.g.*, low K_m_ for perchlorate) (9). Thus, the nature of the residues in the substrate channel in NapA supports the idea of enzyme evolution for a particular function (*i.e.*, nitrate vs perchlorate reduction).

### Chlorate and perchlorate reduction by WT and NapA C176D variant

Steady-state activity measurements indicate that both the WT and NapA C176D variant can accept chlorate and perchlorate as substrates. However, the rate of catalytic turnover for both chlorate and perchlorate reduction is lower for the C176D variant than for WT NapA. This trend is also observed for nitrate reduction. This observation is consistent with a previous observation showing that nitrate reduction by [W(QC_6_H_2_-2,4,6-^i^Pr_3_) (S_2_C_2_Me_2_)]^-^ is faster when Q = S than when Q = O (40). The decrease in k_cat_ of nitrate reduction upon replacement of S of cysteine with O of either Asp or Ser has been reported before (11). The kinetic and energetic results showed that the coordinating Cys residue in WT NapA leads to a lower activation energy for nitrate reduction than Asp or Ser. Thus, it was suggested that the coordinating residue is one component that nature uses to tune substrate electivity. Furthermore, the detrimental effect of C176D on the apparent efficacy of the substrate conversion may be a result of the less selective ion channel in the variant, which, on the one hand, enhances the chance for effective binding of the perchlorate and chlorate but results in a loss of affinity for the native substrate.

### Perchlorate and chlorate reduction by *Cj*NapA and *Ao*PcrAB

Steady-state activity measurements and ESI-MS results indicate that NapA can reduce perchlorate to chlorate (with a small amount of chlorite) and chlorate to chlorite. These results suggest that the perchlorate reduction to chlorite proceeds *via* chlorate as proposed for PcrA (9). NapA-catalyzed chlorate reduction has a k_cat_ (2.1 s^−1^) which is comparable to that of perchlorate reduction (2.5 s^−1^) (Table 1). However, both values are higher than the k_cat_ for perchlorate reductase (0.45 s^−1^). This could be due to the difference in the reductant used during the steady-state kinetic measurements: NADH (−320 mV) for PcrAB vs. methyl viologen (−450 mV) for NapA. Another difference is that the perchlorate reductase activity assays were performed with heterodimeric PcrAB, which can contribute to the sluggish k_cat_. The K_m_ value of *Cj*NapA for chlorate (∼1 mM) is much lower than that for perchlorate (∼23 mM). This lower K_m_ for chlorate can be attributed to the smaller size of chlorate (van der Waal volume ∼40 Å^3^) compared to perchlorate (van der Waals volume ∼61 Å^3^) (41). However, these K_m_ values are much higher than those of *A. oryzae* PcrAB for chlorate (7.4 μM), again supporting the evolution of NapA to utilize a smaller nitrate ion as substrate. On the other hand, PcrA, for which the native substrate is perchlorate, has a wider substrate channel, and thus, it has a similar K_m_ for substrates like chlorate, perchlorate, *etc.*

### Catalytic parameters and microorganisms

The results presented herein show that *Cj*NapA can reduce chlorate and perchlorate to chlorite. However, the catalytic efficiency of the (per)chlorate reduction by *Cj*NapA is much lower than that of nitrate reduction by the same enzyme (Table 1). Previously, it was reported that *Ao*PcrA can reduce nitrate (9). However, the catalytic efficiency of nitrate reduction by *Ao*PcrA is much lower than that of nitrate reduction by *Cj*NapA (Table 1). These results suggest that NapA has evolved to reduce nitrate. In contrast, the catalytic efficiency of the (per)chlorate reduction by *Ao*PcrA is higher than that of nitrate reduction by the same enzyme (Table 1). In addition, the catalytic efficiency of the (per)chlorate reduction by *Ao*PcrA is higher than that of the (per)chlorate reduction by *Cj*NapA (Table 1). These results suggest that PcrA evolved to utilize (per)chlorate as a substrate. Finally, because chlorite is toxic, microorganisms have developed a method to remove chlorite using chlorite dismutase. In this detoxification mechanism, chlorite is converted to chloride and O_2_. This pathway is observed in dissimilatory perchlorate-reducing bacteria (42), in which the chlorite dismutase gene is present in a gene cluster containing perchlorate reductase. For example, in *A. Oryzae,* the perchlorate reductase gene (WP_014235273.1), and the chlorite dismutase (WP_014235269.1) are present in the same gene cluster. However, the gene equivalent to WP_014235269.1 was not found in *C. jejuni*. Thus, from an organismal point of view, the reduction of perchlorate by NapA is detrimental to *C. jejuni*. Therefore, *Cj*NapA will experience selective pressure to have a low affinity for perchlorate or chlorate. Further, the ability of *Cj*NapA to reduce chlorate and perchlorate in the absence of a chlorite dismutase may indicate an unknown mechanism of chlorite reduction.

### Evolutionary implications of NapA’s efficient discrimination between (per)chlorate and nitrate

(Per)chlorate concentrations are highest in arid and semi-arid environments on the modern Earth, and these chlorine oxyanions frequently co-occur with nitrate in these environments (albeit at molar ratios that are always in favor of the nitrogen oxyanion) (43, 44). This observation, along with the well-documented ability of respiratory nitrate reductase (NarG) homologs to catalyze (per)chlorate reduction, spurred Youngblut *et al.* (9) to search for structural features (remnants of selective pressures) in PcrA that give it a greater affinity for (per)chlorate than NarG to preclude organisms without chlorite dismutase from generating the toxic chlorine oxyanion by way of NarG. Although K_m_ cannot be used to define substrate affinity, it can be considered an approximate measure of substrate affinity, with a lower K_m_ indicating higher affinity and *vice versa*. A central mechanism invoked by Youngblut *et al.* is the presence of several aromatic amino acid residues conserved in PcrA homologs that are not observed in NarG homologs. Our studies provide the first enzymological data demonstrating that NapA is bedeviled by the same capacity for promiscuous (per)chlorate reduction despite having a remarkably different active site geometry than NarG. Given that NapA may function as a respiratory nitrate reductase in several organisms, it indicates that NapA has also undergone selective pressures to preclude NapA from efficiently binding (per)chlorate oxyanions over nitrate *in vivo* in natural environments, mechanisms quite distinct from those postulated by Youngblut *et al.* (9).

## Conclusion

We report the first structure of NapA from an ε-proteobacterium and highlight the structural differences between *Cj*NapA and NapA from other classes (α, β, γ, and δ) of proteobacteria. The reported structures reveal several polypeptide insertions that may enhance substrate affinity. Additionally, steady-state activity measurements show that *Cj*NapA can reduce both perchlorate and chlorate in addition to nitrate. Notably, the K_m_ values of *Cj*NapA for these two substrates are higher compared to those for nitrate. MD simulations support the idea that polypeptide insertions on the surface can act as a docking site for nitrate. Overall, our structural, kinetic, phylogenetic analyses, and MD simulations suggest that *Cj*NapA has adapted to exhibit high affinity for nitrate.

The structural comparison between *Cj*NapA and other mononuclear molybdenum-containing enzymes suggests a role for the proximal pterin in electron transfer to the Mo center. Thus, our work lays the groundwork for future studies to confirm the roles of the two pterin moieties in the DMSOR family of enzymes.

## Experimental procedures

### Expression and purification of *Cj*NapA and NapA C176D variant

The expression and purification of *Cj*NapA were performed following a previously reported protocol with the modification of substituting the N-terminal TAT signaling peptide from *C. jejuni* with that from *E. coli* (13). This change did not significantly affect catalytic efficiency; therefore, previously reported values were used. Site-directed mutagenesis was conducted to obtain the *napA* C176D variant using the previously described procedure (11). The expression and purification of NapA C176D variant were performed using the previously described method (11, 13).

### Cryo-EM grid preparation and data acquisition

For grid preparation, a monodisperse fraction of the NapA protein was used. UltraAu grids (R1.2/1.3 mm, 300 mesh) were glow-discharged for 60 s at 25 mA with an additional 10-s hold. Following this, 3.0 μl of the 1.2 mg/ml protein sample was applied to the grids and blotted for 2 s at 4 °C and 100% humidity. Vitrification was achieved by plunging the grids into liquid ethane, cooled by liquid nitrogen, using Vitrobot Mark IV (Thermo Fisher Scientific).

Data were acquired on a Titan Krios (Thermo Fisher Scientific) operating at 300 kV with a nominal magnification of 105,000 × for all datasets. Image acquisition was performed using the automated program SerialEM (45). Micrographs were captured with nominal defocus values ranging from 0.8 to −2.0 μm. A calibrated pixel size of 0.4125 Å was used during the processing. Video frames were recorded using Leginon 3.6 (46) at a dose rate of 28.56 e−/Å^2^/s, with a total exposure time of 1.80 s, resulting in a cumulative dose of 56.8 e−/Å^2^.

### Electron microscopy data processing

The image processing and structure determination workflow consisted of several key steps. Motion correction and image summation were performed using MotionCor2 (47). The contrast transfer function (CTF) parameters for each non-dose-weighted micrograph were estimated, with defocus values determined using Gctf (48). Particle picking was carried out with DoG Picker (49), followed by initial reference-free 2D classification in CryoSPARC v.4.2.0 (50). Representative 2D class averages were selected, and further particle refinement was performed through multiple rounds of 2D classification.

An initial *ab initio* reconstruction was generated in CryoSPARC, and the particles were then classified into five groups using 3D classification, with the initial reconstruction low-pass filtered to 20 Å as the reference model. Several rounds of 3D classification were conducted, and the best particle subset, which showed clear structural features, underwent heterogeneous refinement. The refined particles were further processed using homologous and non-uniform refinement, which produced a map with global resolution determined by the Fourier shell correlation at the 0.143 criterion. The final map was calculated with C1 symmetry in CryoSPARC, and the local resolution was assessed using Phenix (51) and visualized using ChimeraX (52). Final statistical data are provided in Table 2, and a detailed data processing pipeline is outlined in Supporting Information Figure S1.

**Table 2:** Cryo-EM data collection, refinement, validation and statistics

Data collection and processing	xxx	Model composition	xxx
Magnification	105,000	Non-hydrogen atoms	7240
Voltage (kV)	300	Protein residues	889
Electron exposure ((e^–^/Ǻ^2^)	56.8	Ligand: SF4	1
Defocus range ((μm)	0.8–2.0	Mo	1
Raw pixel size (Ǻ)	0.411	MGD	2
Symmetry imposed	C1	**B factors (Ǻ^2^)**	
No of initial particle images	199,859	Protein	78.37
Number of final particle images	131,448	Ligand	61.98
Map resolution (Ǻ)	3.04	**RMSD values**	
FSC threshold	0.123	Bond lengths (Ǻ)	0.003
Map resolution range (Ǻ)	2.5–4.0	Bond angles (^o^)	0.690
Refinement		**Validation**	
Initial model used	Alphafold model	Molprobity score	2.46
Model resolution (Ǻ)	N/A	Clash score	17.83
FSC threshold	0.5	Poor rotamer (%)	2.93
Model resolution range (Ǻ)	N/A	**Ramachandran plot (%)**	
		Favored	94.81
		Allowed	4.96
		Outliers	0.23

### Model building and structure determination

The initial apo-state model of NapA was built in Coot v0.8.9 (53), using the AlphaFold structure as a reference. The initial model was fitted into the EM maps using ChimeraX, followed by iterative manual adjustments and rebuilding in Coot. Throughout the model-building process, refinements were performed based on the quality of the EM maps, with subsequent real-space refinement conducted in Phenix.

The refined model was validated using FSC curves to assess the agreement between the final model and the map. The geometric quality of the atomic models was evaluated using MolProbity (54). The detailed refinement statistics are provided in Table 2. Pore-lining surfaces and channels within the protein were identified using MOLE (55). All visualizations were generated using UCSF ChimeraX and PyMOL.

### Measurement of perchlorate reductase activity

Perchlorate reductase activity of both WT and NapA C176D variant was measured using a procedure similar to that reported for nitrate reductase (13). In brief, the enzyme reaction was initiated by adding perchlorate in place of nitrate, and the activity was measured spectrophotometrically by monitoring the oxidation of reduced methyl viologen at 600 nm. However, the control experiments without any enzyme also showed a small background oxidation of reduced methyl viologen (see Supporting Information, Fig. S2), which was corrected for each substrate concentration. For WT NapA assays, the enzyme concentration was 67 nM, and the substrate concentrations range was 9.4 mM to 383 mM. For the NapA C176 A variant, the enzyme concentration was 217 nM, substrate concentration range 4.68 mM to 150 mM. All assays were conducted in 50 mM HEPES (pH 7) at room temperature inside an argon atmosphere glove box.

### Measurement of chlorate reductase activity

The chlorate reductase activity of the WT and NapA-C176D variant was measured using the method described above for perchlorate, except that the enzyme reaction was initiated by adding chlorate instead of perchlorate. In this case, no significant oxidation of methyl viologen was observed in the absence of the enzyme sample (Supporting Information, Fig. S3). For WT NapA assays, the enzyme concentration was 67 nM and the substrate concentrations range was 0.1 mM to 100 mM. For the NapA C176 A variant, the enzyme concentration was 430 nM, substrate concentration range 0.05 mM to 12.5 mM. All assays were conducted in 50 mM HEPES (pH 7) at room temperature inside an argon atmosphere glove box.

### Detection of products of WT NapA catalyzed reduction of perchlorate and chlorate by mass spectrometry

The detection of products in the enzyme reactions was performed using an electrospray ionization -Thermo Q-Exactive Focus mass spectrometer (Thermo Scientific Inc., San Jose, CA, USA). A negative polarity was used, and the spray voltage was 3.3 kV. The S-lens voltage was 60.0 V, the maximum injection time was 200 ms, the AGC target was 3E6, the capillary temperature was 320 °C, the in-source collision-induced dissociation was 15.0 V, the resolution was 70,000 FWHM, and the raw data were extracted from Xcalibur (Thermo Scientific) and plotted in Origin.

### Inhibition of nitrate reductase activity of WT NapA by perchlorate

Nitrate reductase activity of NapA was measured using a reported procedure (13). For inhibition experiments, the enzyme was incubated with reduced methyl viologen and different concentrations (2.5 mM or 5 mM) of sodium perchlorate for 10 min before the addition of substrate.

### Phylogenetic analysis

The well-characterized NapA homolog from *D. desulfuricans* ATCC 27774 (17) was used as the BLAST query to identify potential NapA homologs across the diversity of prokaryotic life. For comparative genomic analyses, DELTA-BLAST (56) searches were conducted, and homologs were selected only if the sequence originated from an organism isolated in pure culture or defined co-culture, aligned over at least 95% of the query with a minimum amino acid identity of 30%, and if the sequence length was consistent with that of the query. Additionally, the primary sequence had to contain motifs characteristic of the enzyme family, such as a twin-arginine translocation motif, a [4Fe4S] cluster binding motif, and a Mo/W-*bis-*PDT binding motif. Candidates were further screened using the Integrated Microbial Genomics (IMG) platform (57) to examine the genomic context of each putative homolog. Sequences were retained only if the primary sequences from the NCBI database and the IMG database were conserved, and if the operon contained other subunits consistent with the operon structure described in model organisms, such as a [4Fe4S] cluster-containing protein, a [2Fe2S] Rieske protein, and a membrane anchor. This process resulted in 251 NapA homologs for phylogenetic analysis.

Sequences were aligned using the online platform MAFFT for large-scale sequence alignments (58). The resulting alignment was trimmed such that all columns with gaps in more than 20% of NapA sequences or with a similarity score below 0.001 were omitted, with the caveat that 60% of the columns were conserved for the analysis. The amino acid selection model, LG + G4, that best fits the data was chosen using the ModelFinder program (59). Maximum likelihood phylogenies were generated using IQTREE (60). All phylogenies generated using the UltraFast bootstrap approximation were run for 10,000 replicates. All phylogenetic analyses were performed on a laptop computer. The NapA phylogeny was visualized using the Interactive Tree of Life (iTOL) program (61). Well-characterized formate dehydrogenases (FDHs) from *Moorella thermoacetica* (62), *Peptoclostridium acidaminophilum* (63), *E. coli* (64), *Methanococcus maripaludis* (65), and *Methanococcus vannielii* (66) were selected as outgroups since our previous work has found NapA to be an evolutionary descendant of this group (3).

DELTA-BLAST searches were also used to screen the genomes of organisms whose NapA homologs were included in our phylogenetic analysis for perchlorate reductase, chlorate reductase, and chlorite dismutase. All sequence identity and query length requirements were identical to the criteria for the NapA homolog search. The perchlorate reductase of *M. thermoacetica* (43), chlorate reductase of *Ideonella dechloratans* (67), chlorite dismutase of *Dechloromonas aromatica* (68), and chlorite dismutase of *Nitrobacter winogradsky* (69) were selected as queries to execute DELTA-BLAST searches for (per)chlorate metabolism.

### MD simulation

The final apo-state model of NapA derived from cryo-EM was used as a starting point for model construction and used in molecular dynamics simulations. The Moco cofactor was modified to a hexacoordinate form with the addition of a water ligand, following the geometry obtained in our QM:MM study (39). The model was protonated by H++ at pH 7.0 and 0.2 M salt concentration, while the charge of the model was neutralized by the addition of 8 Cl^-^ ions. The simulation was conducted in a TIP3P water box, following the previously described protocol. The parameters for Moco and substrates (perchlorate, chlorate, or nitrate) were either derived by DFT and antechamber procedure as described previously (39, 70), or taken from the AMBER parameter database (www.amber.manchester.ac.uk) (71, 72). The details on parametrization procedures are in Supporting Information. The following simulations were conducted using AMBER24 for WT NapA and Cys176Asp NapA variant: 100 ns apoenzyme (in triplicate), 100 ns enzyme-substrate complex (NO_3_^-^, ClO_3_^-^, ClO_4_^-^) for a hexacoordinate Mo-center and a pentacoordinate Mo-center (without the coordinated water ligand), 100 to 150 ns enzyme at 200 mM concentration of NaNO_3,_ NaClO_3_ or NaClO_4_ for both hexa and pentacoordinate forms. The initial positions of the 115 ions in the simulation box (105 × 95.4 × 95.8 Å^3^) were obtained using the PACKMOL package (73). The calculated trajectories were analyzed for distances of ions from the Mo-center or selected Lys-Nζ atoms, root-mean square fluctuations (RMSF) and deviations (RMSD) calculated for the main chain and active site heavy atoms (see SI for the mask), interaction energies between ion ligand and protein residues, and MM/PBSA ΔG of binding using the Generalized Born approach.

## Data availability

All data supporting the findings of this study are included within the article and its Supporting Information. Structural coordinates and structure factors for the *C. jejuni* periplasmic nitrate reductase (NapA) have been deposited in the Protein Data Bank (9PXT). Additional raw data, including enzyme kinetics and substrate profiling results, are available from the corresponding author upon reasonable request.

## Supporting information

This article contains supporting information (71, 74, 75, 76, 77, 78, 79, 80, 81).

## Conflicts of interest

The authors declare that they have no conflicts of interest with the contents of this article.
