# Supplementary material for: Structure and substrate promiscuity of Campylobacter jejuni periplasmic nitrate reductase (Nap) and phylogenetic analysis of Nap homologs
Source: J Biol Chem. 2025 Nov 15;301(12):110928. doi: 10.1016/j.jbc.2025.110928 (PMC12769810; doi:10.1016/j.jbc.2025.110928)
Supplement: Supporting information [file mmc1.docx]

Structure, evolution and substrate promiscuity of Periplasmic Nitrate Reductase.

Nitai C. Giri, Trung Thach, Kanaga Vijayan Dhanabalan, Mintare Cesiunaite, Manohar Radhakrishnan, Lahiru Wedasingha, Nicholas Manicke, Micheal Wells, Maciej Szaleniec, Ramaswamy Subramanian, Partha Basu

Supporting information

Table of contents

Figure S1. ……………………………………………………………………………………………………………………………… S2

Figure S2. ……………………………………………………………………………………………………………………………… S3

Figure S3. ……………………………………………………………………………………………………………………………… S4

Figure S4. ……………………………………………………………………………………………………………………………… S5

Figure S5. ……………………………………………………………………………………………………………………………… S6

Figure S6. ………………………………………………………………………………………………………………….…………… S7

Figure S7. ………………………………………………………………………………………………………………….…………… S8

Figure S8. ………………………………………………………………………………………………………………….…………… S9

Figure S9. ………………………………………………………………………………………………………………….…………… S10

Figure S10. ………………………………………………………………………………………………………………….………… S11

Figure S11. ………………………………………………………………………………………………………………….………… S12

Figure S12. ………………………………………………………………………………………………………………….………… S13

Figure S13. ……………………………………………………………………………………………………………………………… S14

Figure S14. ……………………………………………………………………………………………………………….…………… S15

Figure S15. ……………………………………………………………………………………………………………….…………… S16

Figure S16. ……………………………………………………………………………………………………………….…………… S17

Figure S17. ……………………………………………………………………………………………………………….…………… S18

Figure S18. ……………………………………………………………………………………………………………….…………… S19

Figure S19. ……………………………………………………………………………………………………………….…………… S20

Figure S20. ……………………………………………………………………………………………………………….…………… S21

Figure S21. ……………………………………………………………………………………………………………….…………… S22

MD model preparation…………………………………………………………………………………………….…………… S22

MD simulation…………………………………………………………………………………………….……………………..… S23

Figure S1. Cryo-EM data processing pipeline


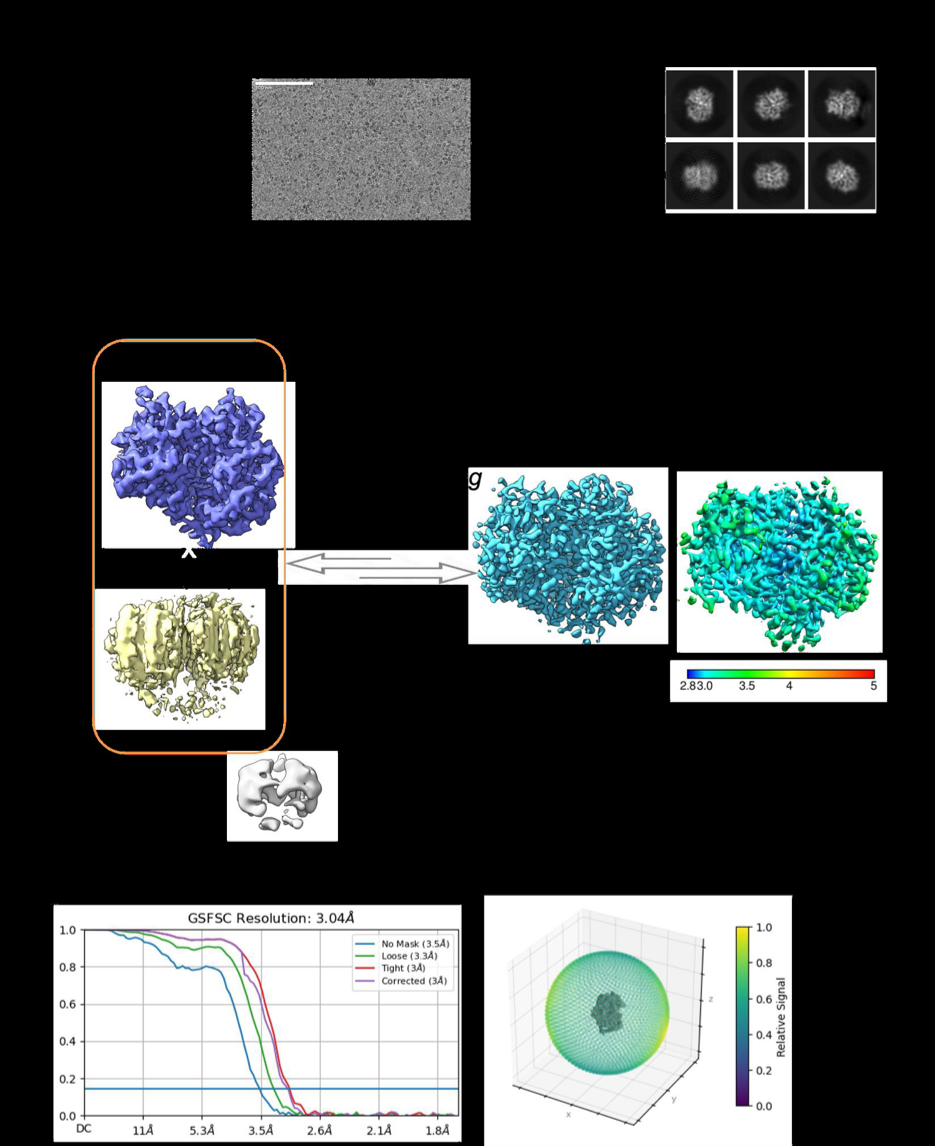


Figure S2. No enzyme control shows a background reaction during perchlorate reduction by *Cj*NapA that has been corrected in reported measurements.

Figure S3. No enzyme control does not show a significant background reaction during chlorate reduction by *Cj*NapA

Figure S4. Conserved lysine in between the proximal pterin and [4Fe4S] cluster in NapA from *C. jejuni* (green), *C. sphaeroides* (cyan) and *E. coli* (gray)


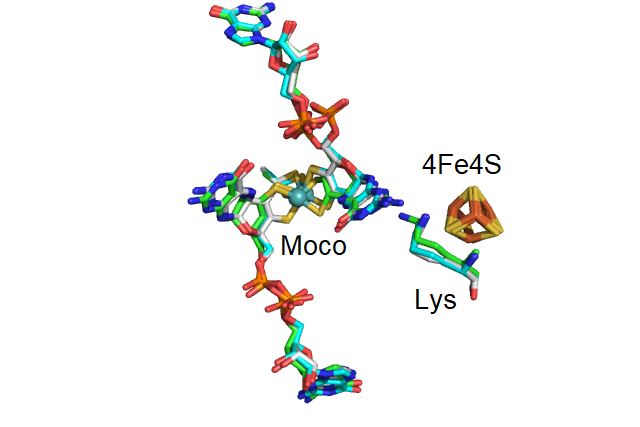


Figure S5. Results from MD simulation for NapA with hexacoordinate Moco and 0.02 M NaNO_3_; RMSD of the WT hexacoordinate Moco and B) C176D hexacoordinate Moco (black – main chain, red – active site); C) Radial Distribution Function of Mo-NO_3_ distance, D) statistical parameters of minimum distance of Mo-NO_3_

| A) | B) |
| --- | --- |
| C) | D)   \| Min Mo-NO3 \| Mean \| SD \| Minimum \| Median \| Maximum \| \| --- \| --- \| --- \| --- \| --- \| --- \| \| WT \| 20.33 \| 2.84 \| 14.61 \| 20.37 \| 31.64 \| \| C176D \| 19.89 \| 3.01 \| 11.55 \| 20.11 \| 30.07 \| |

Figure S6. Results from MD simulation for NapA with pentacoordinate Moco and 0.02 M NaNO_3_; RMSD of the WT pentacoordinate Moco and B) C176D pentacoordinate Moco (black – main chain, red – active site); C) Radial Distribution Function of Mo-NO_3_ distance, D) statistical parameters of minimum distances of Mo-NO_3_

| A)  | B)  |
| --- | --- |
| C)  | D)   \| Min Mo-NO3 \| Mean \| SD \| Minimum \| Median \| Maximum \| \| --- \| --- \| --- \| --- \| --- \| --- \| \| WT \| 19.87 \| 3.22 \| 11.44 \| 20.60 \| 27.95 \| \| C176D \| 19.87 \| 3.22 \| 11.44 \| 20.60 \| 27.95 \| |

Figure S7. Results from MD simulation for NapA with hexacoordinate Moco and 0.02 M NaClO_3_; RMSD of the WT hexacoordinate Moco and B) C176D hexacoordinate Moco (black – main chain, red – active site); C) Radial Distribution Function of Mo-ClO_3_ distance, D) statistical parameters of minimum distance of Mo-ClO_3_

| A)   | B)  |
| --- | --- |
| C)  | D)   \| Min Mo-ClO_3_ \| Mean \| SD \| Minimum \| Median \| Maximum \| \| --- \| --- \| --- \| --- \| --- \| --- \| \| WT \| 20.09 \| 2.47 \| 11.72 \| 20.23 \| 27.37 \| \| C176D \| 20.94 \| 2.47 \| 9.29 \| 21.10 \| 25.99 \| |

Figure S8. Results from MD simulation for NapA with pentacoordinate Moco and 0.02 M NaClO_3_; RMSD of the WT pentacoordinate Moco and B) C176D pentacoordinate Moco (black – main chain, red – active site); C) Radial Distribution Function of Mo-ClO_3_ distance, D) statistical parameters of minimum distance of Mo-ClO_3_

| A)   | B)  |
| --- | --- |
| C)  | D)   \| Min Mo-ClO_3_ \| Mean \| SD \| Minimum \| Median \| Maximum \| \| --- \| --- \| --- \| --- \| --- \| --- \| \| WT \| 19.85 \| 2.60 \| 12.72 \| 20.13 \| 25.86 \| \| C176D \| 20.74 \| 2.70 \| 11.23 \| 20.88 \| 27.49 \| |

Figure S9. Results from MD simulation for NapA with hexacoordinate Moco and 0.02 M NaClO_4_; RMSD of the WT hexacoordinate Moco and B) C176D hexacoordinate Moco (black – main chain, red – active site); C) Radial Distribution Function of Mo-ClO_4_ distance, D) statistical parameters of minimum distance of Mo-ClO_4_

| A)    | B)  |
| --- | --- |
| C)  | D)   \| Min Mo-ClO_3_ \| Mean \| SD \| Minimum \| Median \| Maximum \| \| --- \| --- \| --- \| --- \| --- \| --- \| \| WT \| 20.16 \| 2.43 \| 0.23 \| 12.68 \| 20.27 \| \| C176D \| 20.70 \| 1.88 \| 0.18 \| 11.22 \| 20.65 \| |

Figure S10. Results from MD simulation for NapA with hexacoordinate Moco and 0.02 M NaClO_4_; RMSD of the WT hexacoordinate Moco and B) C176D hexacoordinate Moco (black – main chain, red – active site); C) Radial Distribution Function of Mo-ClO_4_ distance, D) statistical parameters of minimum distance of Mo-ClO_4_

| A)    | B)  |
| --- | --- |
| C)  | D)   \| Min Mo-ClO_3_ \| Mean \| SD \| Minimum \| Median \| Maximum \| \| --- \| --- \| --- \| --- \| --- \| --- \| \| WT \| 20.83 \| 2.51 \| 11.62 \| 20.77 \| 26.87 \| \| C176D \| 20.79 \| 2.88 \| 12.23 \| 20.81 \| 32.35 \| |

Figure S11. Difference between RMSF of pentacoordinate and hexacoordinate apo NapA for A) WT B) C176D and difference between RMSF of C176D and WT mutant apo NapA for C) hexacoordinate and D) pentacoordinate Moco. The red regions indicate increased flexibility (positive ΔB), blue regions indicate decreased flexibility (negative ΔB). Both figures are on the same scale (ΔB factors range from -15 to 35)

| A | B |
| --- | --- |
| 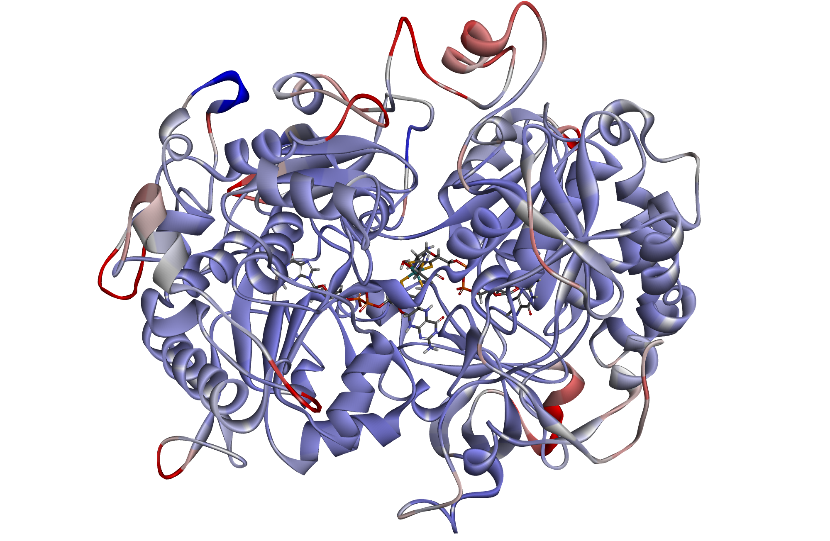 | 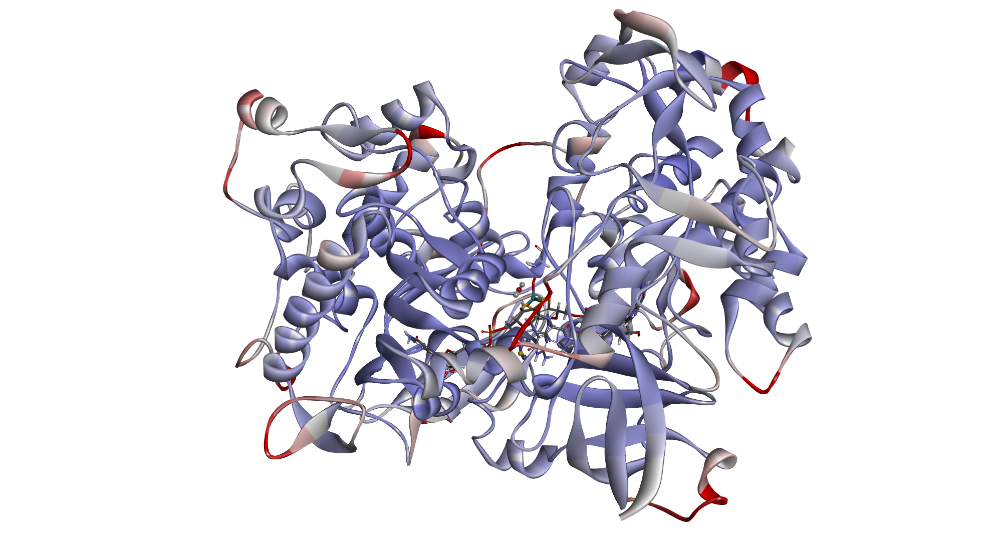 |
| C | D |
| 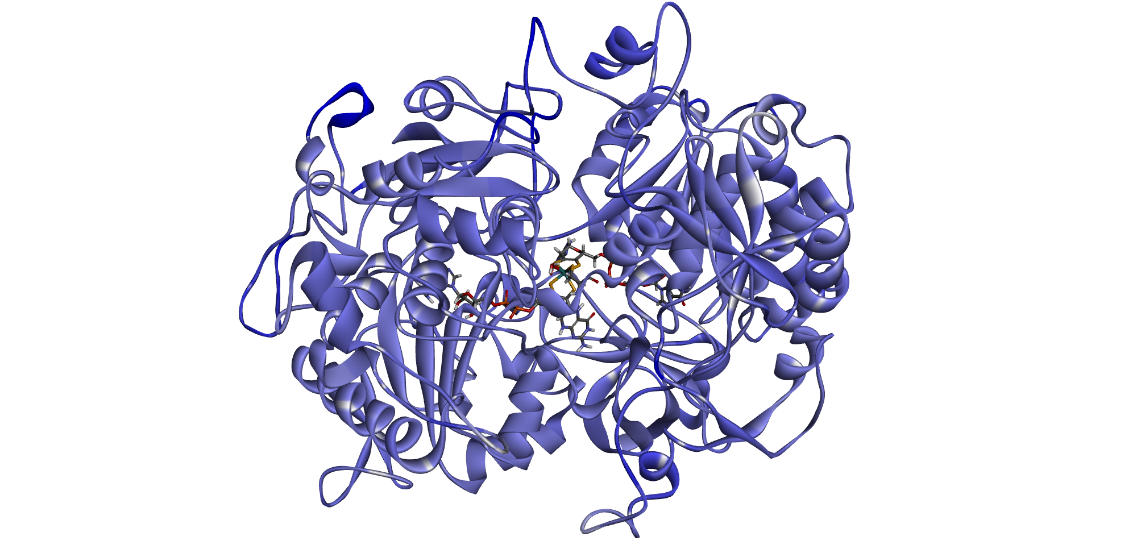 | 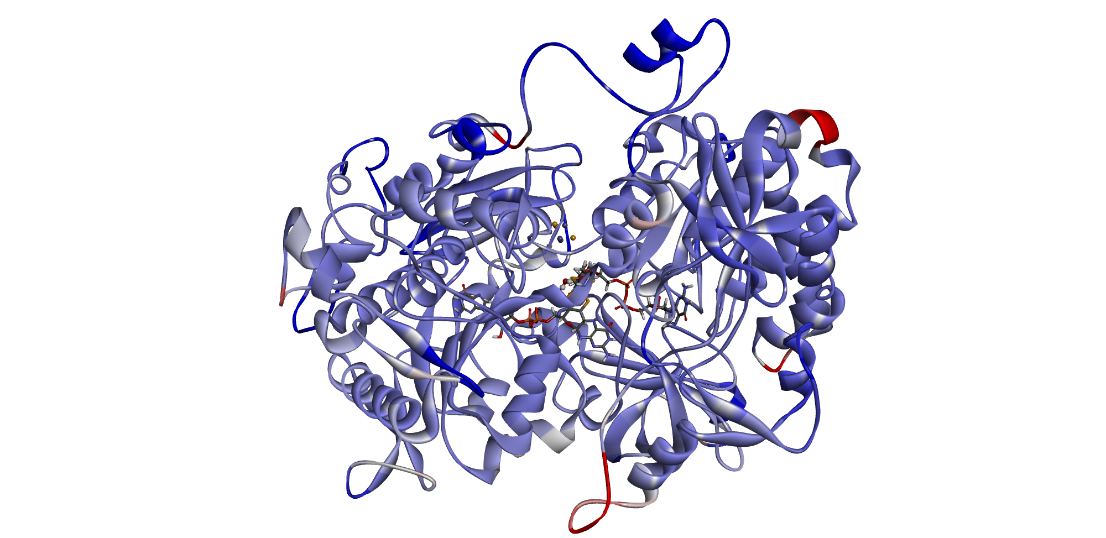 |

Figure S12. Results from MD simulation for NapA with hexacoordinate Moco and bound NO_3_^-^ for the WT and C176D mutant; A and B RMSD of the main chain atoms - black curve and active site (Moco and residues surrounding the nitrate) - red curve; C and D distance between Mo and N atom of NO_3_^-^ and its histograms (E and F respectively) followed by statistical data for the Mo-N distances.

| NAP WT | NAP C176D |
| --- | --- |
| A  | B  |
| C  | D  |
| E  | F  |

| Data | Mean | Standard Deviation | SE of mean | Minimum | Median | Maximum |
| --- | --- | --- | --- | --- | --- | --- |
| WT Mo-N [Å] | 8 | 0.31 | 9.6E-04 | 4.98 | 8.01 | 9.17 |
| C176D Mo-N [Å] | 13 | 2.0 | 0.00642 | 4.64 | 12.57 | 18.31 |

Figure S13. Results from MD simulation for NapA with pentacoordinate Moco – bound NO_3_^-^ for the WT and C176D mutant; A and B RMSD of the main chain atoms - black curve and active site (Moco and residues surrounding the nitrate) - red curve; C and D distance between Mo and N atom of NO_3_^-^ and its histograms (E and F respectively) followed by statistical data for the Mo-N distances.

| NAP WT | NAP C176D |
| --- | --- |
| A  | B  |
| C  | D  |
| E  | F  |

| Data | Mean | Standard Dev | SE of mean | Minimum | Median | Maximum |
| --- | --- | --- | --- | --- | --- | --- |
| WT Mo-N [Å] | 9.0 | 0.46 | 0.0015 | 4.78 | 8.97 | 12.42 |
| C176D Mo-N [Å] | 33.5 | 16.7 | 0.05 | 4.82 | 32.27 | 77.8 |

Figure S14. Results from MD simulation for NapA with hexacoordinate Moco – bound ClO_3_^-^ for the WT and C176D mutant; A and B RMSD of the main chain atoms - black curve and active site (Moco and residues surrounding the nitrate) - red curve; C and D distance between Mo and Cl atom of ClO_3_^-^ and its histograms (E and F respectively) followed by statistical data for the Mo-Cl distances.

| NAP WT | NAP C176D |
| --- | --- |
| A  | B  |
| C  | D  |
| E | F |

| Data | Mean | Standard Dev | SE of mean | Minimum | Median | Maximum |
| --- | --- | --- | --- | --- | --- | --- |
| WT Mo-Cl [Å] | 7.88 | 1.32 | 0.0042 | 4.90 | 8.22 | 12.35 |
| C176D Mo-Cl [Å] | 5.23 | 0.41 | 0.0013 | 4.31 | 5.1 | 7.84 |

Figure S15. Results from MD simulation for NapA with pentacoordinate Moco – bound ClO_3_^-^for the WT and C176D mutant; A and B RMSD of the main chain atoms - black curve and active site (Moco and residues surrounding the nitrate) - red curve; C and D distance between Mo and Cl atom of ClO_3_^-^ and its histograms (E and F respectively) followed by statistical data for the Mo-Cl distances.

| NAP WT | NAP C176D |
| --- | --- |
| A  | B  |
| C  | D  |
| E | F |

| Data | Mean | Standard Dev | SE of mean | Minimum | Median | Maximum |
| --- | --- | --- | --- | --- | --- | --- |
| WT Mo-Cl [Å] | 7.64 | 0.26 | 8.2E-04 | 5.79 | 7.64 | 9.03 |
| C176D Mo-Cl [Å] | 8.78 | 1.92 | 0.0060 | 5.56 | 8.32 | 18.41 |

Figure S16. Results from MD simulation for NapA hexacoordinate Moco – bound ClO_4_^-^ for the WT and C176D mutant; A and B RMSD of the main chain atoms - black curve and active site (Moco and residues surrounding the nitrate) - red curve; C and D distance between Mo and Cl atom of ClO_4_^-^ and its histograms (E and F respectively) followed by statistical data for the Mo-Cl distances.

| NAP WT | NAP C176D |
| --- | --- |
| A  | B  |
| C  | D  |
| E  | F  |

| Data | Mean | Standard Dev | SE of mean | Minimum | Median | Maximu |
| --- | --- | --- | --- | --- | --- | --- |
| WT Mo-Cl [Å] | 6.46 | 0.75 | 0.0024 | 4.61 | 6.23 | 10.4 |
| C176D Mo-Cl [Å] | 5.2 | 0.22 | 6.9E-04 | 4.26 | 5.2 | 6.4 |

Figure S17. Results from MD simulation for NapA pentacoordinate Moco – bound ClO_4_^-^ for the WT and C176D mutant; A and B RMSD of the main chain atoms - black curve and active site (Moco and residues surrounding the nitrate) - red curve; C and D distance between Mo and Cl atom of ClO_4_^-^ and its histograms (E and F respectively) followed by statistical data for the Mo-Cl distances.

| NAP WT | NAP C176D |
| --- | --- |
| A  | B  |
| C  | D  |
| E | F |

| Data | Mean | Standard Dev | SE of mean | Minimum | Median | Maximum |
| --- | --- | --- | --- | --- | --- | --- |
| WT Mo-Cl [Å] | 8.90 | 0.86 | 0.00 | 7.18 | 8.60 | 12.75 |
| C176D Mo-Cl [Å] | 15.3 | 7.1 | 0.02 | 4.98 | 13.78 | 29.78 |

Figure S18. Stable binding sites of NO_3_^-^ in WT NapA: A) for hexacoordinate Moco – Mo-N distance of 8 Å; B) for pentacoordinate Moco – Mo-N distance of 9 Å; values under residues represent interaction energies in kcal/mol between substrate and residues

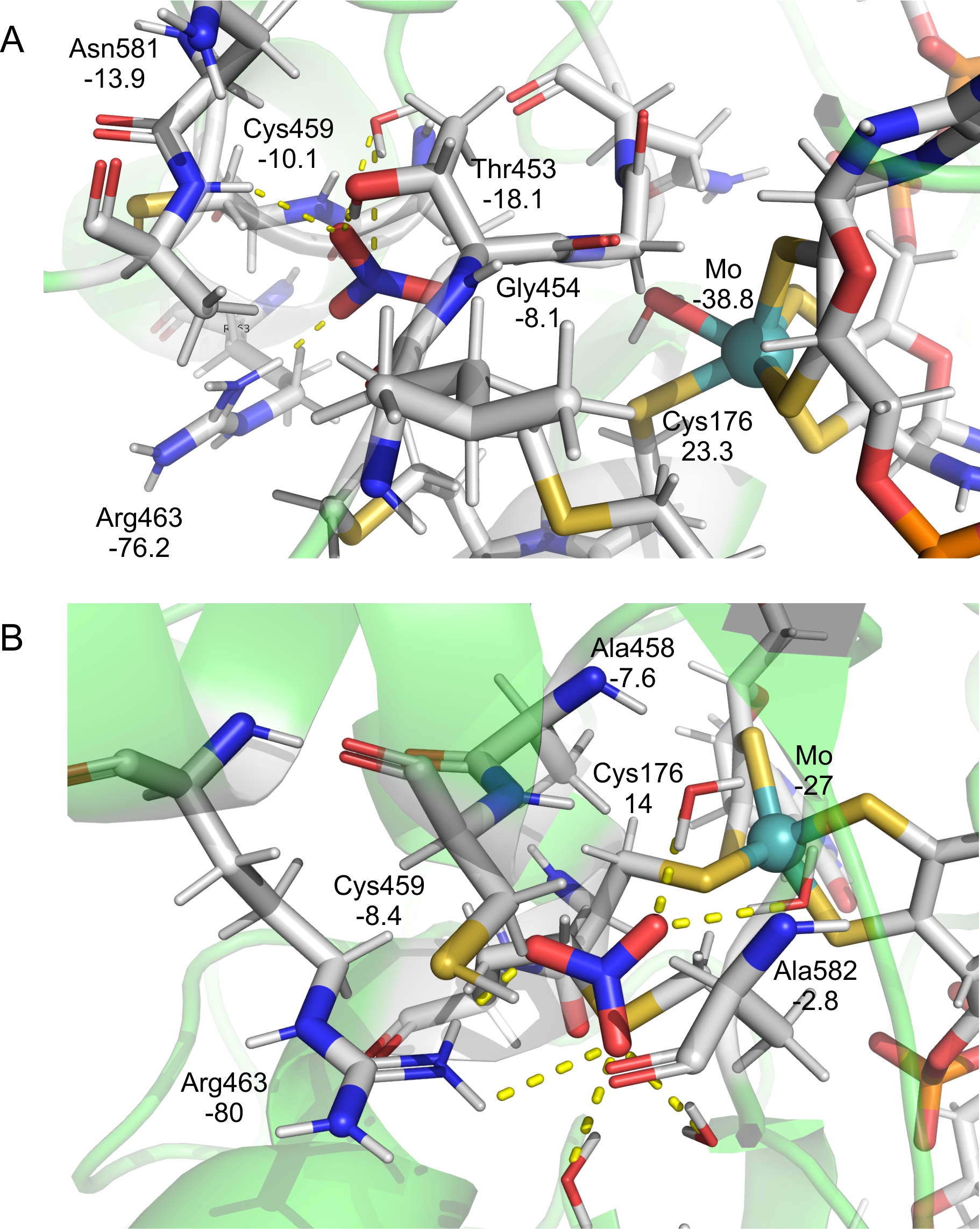


Figure S19. Radial distribution function of NO_3_ ions around Lys residues (NZ positively charged atoms) during WT NapA simulation in 0.02 M NaNO_3_

Figure S20. MM/PBSA estimation of ΔG of binding of ions for MD simulations conducted for WT and C176D NapA hexacoordinate Moco

| WT NO_3_^-^ | C176D NO_3_^-^ |
| --- | --- |
| 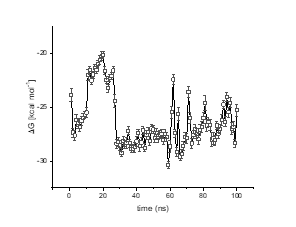 | 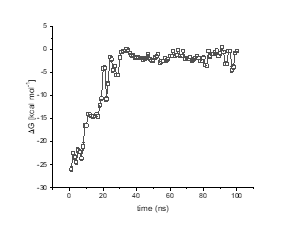 |
| WT ClO_3_^-^ | C176D ClO_3_^-^ |
| 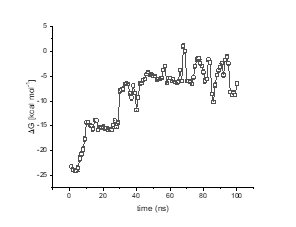 | 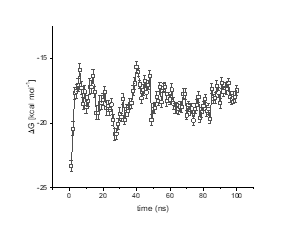 |
| WT ClO_4_^-^ | C176D ClO_4_^-^ |
| 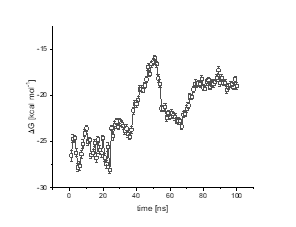 | 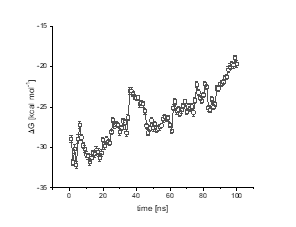 |

Figure S21. Optimized model of MoCo used for parameterization of AMBER parameters.

| A) 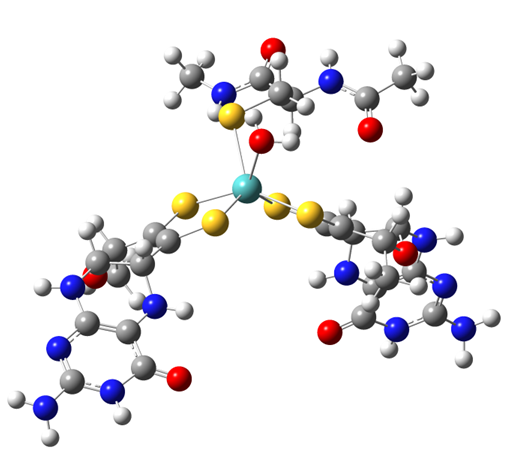 | B) 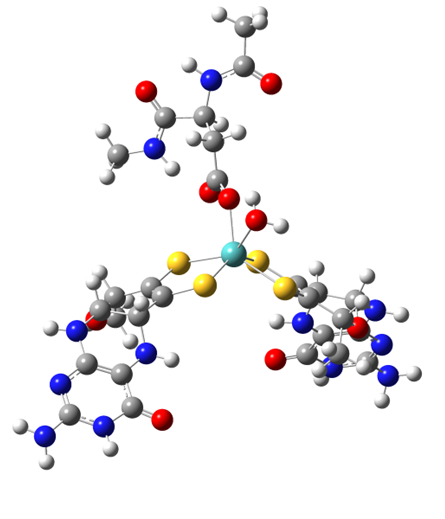 |
| --- | --- |
| C) 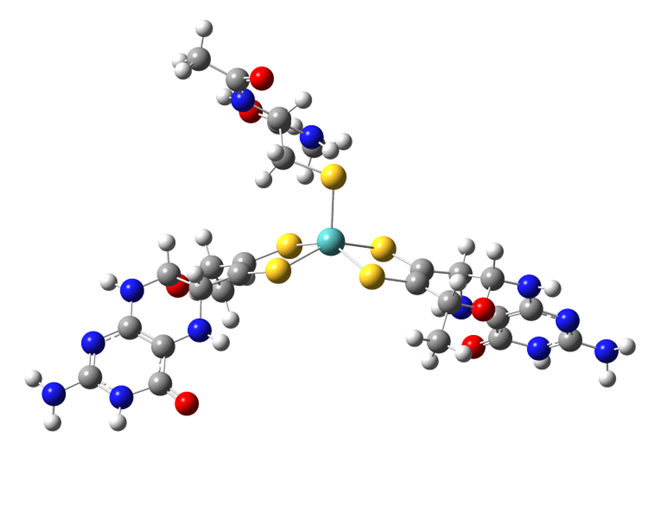 | D) 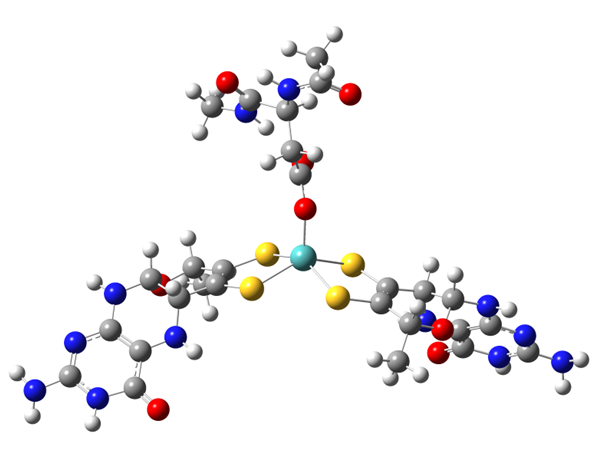 |

**MD model preparation**

**Parametrisation of MoCo.** The missing AMBER parameters for Moco were obtained according to the previously described protocol(1). In short, the geometry of the model MoCo center comprised of Mo(IV), two molybdopterin guanine dinucleotide ligands in a tetrahydropterin form, truncated at the phosphate chains, cysteine or aspartate for pentacoordiante version of MoCo or additionally with bound water for hexacoordiante form of MoCo. The models were optimized in the gas phase in Gaussian16 C.01(2) using B3LYP functional, 6-31g(d,p) for the light atoms and LANL2DZ with pseudopotential for Mo (Figure S22). The force constants for bond stretch and angles were obtained with XYZViever_097 provided courtesy of Sven Marothy. The point charges were obtained using Merz-Kollman electron density calculations(2), followed by the Restrained Electrostatic Potential (RESP) procedure available in the Antechamber of the AmberTools(3). The parameters for the iron-sulfur cluster [4Fe-4S](Cys)_4_ were derived from (4,5) while the lacking non-bonding parameters were derived from (6). The parameters for nitrate (V), perchlorate and chlorate were obtained from the AMBER Parameter Database at Manchester University UK (7) with charges corrected by RESP procedure.

The model of the enzyme was protonated at a pH of 7.0 using H++ using 0.2 M ionic strength and 80-10 range of dielectric constant (water-protein interior). The charge of the system (+8) was neutralised with 8 Cl^-^ ions and the enzyme model was solvated with 1653 explicit tip3p water molecules within a 10 Å radius of the protein. The calculations were conducted in a periodic boundary box (105 Å × 95.4 Å × 95.9 Å). In the case of the models with the bound substrate, 7 Cl^-^ ions were used, while for the simulations in 200 mM solution of the substrate, 115 ions of substrate (NO_3_^-^, ClO_4_^-^ or ClO_3_^-^) were neutralized by 107 of Na^+^. The initial positions of substrate ions were obtained with the packmol program (taking into consideration the position of the enzyme), while sodium ions were added by Leap with the additions command.

The geometry of the model was minimized before the MD simulation according to the following stepwise protocol:

- Minimization of the geometry of water solvent (imin=1, maxcyc=50000, ncyc=2500, ntb=1, ntr=1) with protein residue and Cl^-^ ions frozen with force 500
- Minimization of the geometry of water solvent (imin=1, maxcyc=50000, ncyc=2500, ntb=1, ntr=1) with protein residue and Cl^-^ ions frozen with force 250
- Minimization of the geometry of water solvent and ions (imin=1, maxcyc=10000, ncyc=2500, ntb=1, ntr=1) with protein residue frozen with force 100
- Minimization of the geometry (imin=1, maxcyc=50000, ncyc=2500, ntb=1, ntr=1) with protein residue frozen with force 10
- Minimization of the geometry (imin=1, maxcyc=100000, ncyc=5000, ntb=1, ntr=1) without any constrains

**MD simulations**

All classical MD simulations were performed using the AMBER ff03 force field in AMBER22(8). The heating from 0 to 303 K was performed over 100 ps with the NVT ensemble, and the system was equilibrated during 100 ps with NPT conditions. Subsequently, NPT nonaccelerated and non-biased molecular dynamics (MD) simulations at 303 K were conducted for 100-150 ns using periodic boundary conditions. In total, 3 independent 100 ns simulations were conducted for apo wild type and C176D enzyme variants, along with single 100-150 ns simulations for WT/C176 enzyme in hexa or pentacoordinate version of the cofactor with NO_3_^-^/ClO_3_^-^/ClO_4_^-^ ion bound at the active site or submerged in the 200 mM solution of the respective sodium salts. The simulations were analyzed in terms of RMSD of the main chain and the residue of the active site according to the following mask:

WT:

:146@N,:146@HN,:146@CA,:146@HA,:146@CB,:146@HB3,:146@HB2,:146@SG,:146@C,:146@O,:387@N,:387@H,:387@CA,:387@HA,:387@CB,:387@HB2,:387@HB3,:387@CG,:387@HG2,:387@HG3,:387@SD,:387@CE,:387@HE1,:387@HE2,:387@HE3,:387@C,:387@O,:423@N,:423@H,:423@CA,:423@HA,:423@CB,:423@HB,:423@CG2,:423@HG21,:423@HG22,:423@HG23,:423@OG1,:423@HG1,:423@C,:423@O,:424@N,:424@H,:424@CA,:424@HA2,:424@HA3,:424@C,:424@O,:425@N,:425@H,:425@CA,:425@HA,:425@CB,:425@HB2,:425@HB3,:425@CG,:425@HG2,:425@HG3,:425@CD,:425@OE1,:425@NE2,:425@HE21,:425@HE22,:425@C,:425@O,:786@N,:786@H,:786@CA,:786@HA,:786@CB,:786@HB2,:786@HB3,:786@CG,:786@HG2,:786@HG3,:786@CD,:786@HD2,:786@HD3,:786@NE,:786@HE,:786@CZ,:786@NH1,:786@HH11,:786@HH12,:786@NH2,:786@HH21,:786@HH22,:786@C,:786@O,:896@Mo,:896@O,:897@PA,:897@PB,:897@C5',:897@O5',:897@C4',:897@O4',:897@C3',:897@O3',:897@C2',:897@O2',:897@C1',:897@N1,:897@O1A,:897@O1B,:897@C2,:897@N2,:897@O2A,:897@O2B,:897@N3,:897@O3A,:897@O3B,:897@C4,:897@C5,:897@C6,:897@O6,:897@N7,:897@C8,:897@N9,:897@C10,:897@C11,:897@O11,:897@C12,:897@S12,:897@C13,:897@S13,:897@C14,:897@N15,:897@C16,:897@C17,:897@O17,897@N18,897@C19,897@N19,897@N20,897@C21,897@N22,897@C23,897@H1,897@H2,897@H3,897@H4,897@H5,897@H6,897@H7,897@H8,897@H9,897@H10,897@H11,897@H12,897@H13,897@H14,897@H15,897@H16,897@H17,897@H18,897@H19,897@H20,897@H21,897@H22,898@PA,898@PB,898@C5',898@O5',898@C4',898@O4',898@C3',898@O3',898@C2',898@O2',898@C1',898@N1,898@O1A,898@O1B,898@C2,898@N2,898@O2A,898@O2B,898@N3,898@O3A,898@O3B,898@C4,898@C5,898@C6,898@O6,898@N7,898@C8,898@N9,898@C10,898@C11,898@O11,898@C12,898@S12,898@C13,898@S13,898@C14,898@N15,898@C16,898@C17,898@O17,898@N18,898@C19,898@N19,898@N20,898@C21,898@N22,898@C23

C176 mutant (in MD C176 is numbered as C146)

:146@N,:146@HN,:146@CA,:146@HA,:146@CB,:146@HB3,:146@HB2,:146@CG,:146@OD1,:146@OD2,:146@C,:146@O,:387@N,:387@H,:387@CA,:387@HA,:387@CB,:387@HB2,:387@HB3,:387@CG,:387@HG2,:387@HG3,:387@SD,:387@CE,:387@HE1,:387@HE2,:387@HE3,:387@C,:387@O,:423@N,:423@H,:423@CA,:423@HA,:423@CB,:423@HB,:423@CG2,:423@HG21,:423@HG22,:423@HG23,:423@OG1,:423@HG1,:423@C,:423@O,:424@N,:424@H,:424@CA,:424@HA2,:424@HA3,:424@C,:424@O,:425@N,:425@H,:425@CA,:425@HA,:425@CB,:425@HB2,:425@HB3,:425@CG,:425@HG2,:425@HG3,:425@CD,:425@OE1,:425@NE2,:425@HE21,:425@HE22,:425@C,:425@O,:786@N,:786@H,:786@CA,:786@HA,:786@CB,:786@HB2,:786@HB3,:786@CG,:786@HG2,:786@HG3,:786@CD,:786@HD2,:786@HD3,:786@NE,:786@HE,:786@CZ,:786@NH1,:786@HH11,:786@HH12,:786@NH2,:786@HH21,:786@HH22,:786@C,:786@O,:896@Mo,:896@O,:897@PA,:897@PB,:897@C5',:897@O5',:897@C4',:897@O4',:897@C3',:897@O3',:897@C2',:897@O2',:897@C1',:897@N1,:897@O1A,:897@O1B,:897@C2,:897@N2,:897@O2A,:897@O2B,:897@N3,:897@O3A,:897@O3B,:897@C4,:897@C5,:897@C6,:897@O6,:897@N7,:897@C8,:897@N9,:897@C10,:897@C11,:897@O11,:897@C12,:897@S12,:897@C13,:897@S13,:897@C14,:897@N15,:897@C16,:897@C17,:897@O17,897@N18,897@C19,897@N19,897@N20,897@C21,897@N22,897@C23,897@H1,897@H2,897@H3,897@H4,897@H5,897@H6,897@H7,897@H8,897@H9,897@H10,897@H11,897@H12,897@H13,897@H14,897@H15,897@H16,897@H17,897@H18,897@H19,897@H20,897@H21,897@H22,898@PA,898@PB,898@C5',898@O5',898@C4',898@O4',898@C3',898@O3',898@C2',898@O2',898@C1',898@N1,898@O1A,898@O1B,898@C2,898@N2,898@O2A,898@O2B,898@N3,898@O3A,898@O3B,898@C4,898@C5,898@C6,898@O6,898@N7,898@C8,898@N9,898@C10,898@C11,898@O11,898@C12,898@S12,898@C13,898@S13,898@C14,898@N15,898@C16,898@C17,898@O17,898@N18,898@C19,898@N19,898@N20,898@C21,898@N22,898@C23

Furthermore, the distances of ions from Mo center were analyzed by means of statistical distribution during the stable phase of the dynamics or radial distribution function. The energetics of the interaction of the bound substrate into the active site or pre-binding sites was analyzed by means of MMPBSA(9) or interaction energy. Finally, the flexibility of the studied models was analyzed by means of Root-mean-square-fluctuations (RMSF).

References

1. Rugor, A., Wójcik-Augustyn, A., Niedzialkowska, E., Mordalski, S., Staroń, J., Bojarski, A., and Szaleniec, M. (2017 ) Reaction mechanism of sterol hydroxylation by steroid C25 dehydrogenase – Homology model, reactivity and isoenzymatic diversity. *J. Inorg. Biochem.* **173**, 28–43

2. Frisch, M. J., Trucks, G. W., Schlegel, H. B., Scuseria, G. E., Robb, M. A., Cheeseman, J. R., Scalmani, G., Barone, V., Petersson, G. A., Nakatsuji, H., Li, X., Caricato, M., Marenich, A. V., Bloino, J., Janesko, B. G., Gomperts, R., Mennucci, B., Hratchian, H. P., Ortiz, J. V., Izmaylov, A. F., Sonnenberg, J. L., Williams, Ding, F., Lipparini, F., Egidi, F., Goings, J., Peng, B., Petrone, A., Henderson, T., Ranasinghe, D., Zakrzewski, V. G., Gao, J., Rega, N., Zheng, G., Liang, W., Hada, M., Ehara, M., Toyota, K., Fukuda, R., Hasegawa, J., Ishida, M., Nakajima, T., Honda, Y., Kitao, O., Nakai, H., Vreven, T., Throssell, K., Montgomery Jr., J. A., Peralta, J. E., Ogliaro, F., Bearpark, M. J., Heyd, J. J., Brothers, E. N., Kudin, K. N., Staroverov, V. N., Keith, T. A., Kobayashi, R., Normand, J., Raghavachari, K., Rendell, A. P., Burant, J. C., Iyengar, S. S., Tomasi, J., Cossi, M., Millam, J. M., Klene, M., Adamo, C., Cammi, R., Ochterski, J. W., Martin, R. L., Morokuma, K., Farkas, O., Foresman, J. B., and Fox, D. J. (2016) Gaussian 16 Rev. C.01. Wallingford, CT

3. Salomon-Ferrer, R., Case, D. A., and Walker, R. C. (2013) An overview of the Amber biomolecular simulation package. *Wiley Interdisciplinary Reviews: Computational Molecular Science* **3**, 198-210

4. Carvalho, A. T. P., and Swart, M. (2014) Electronic Structure Investigation and Parametrization of Biologically Relevant Iron–Sulfur Clusters. *J Chem Inf Model* **54**, 613-620

5. Carvalho, A. T. P., and Swart, M. (2015) Correction for Electronic Structure Investigation and Parametrization of Biologically Relevant Iron–Sulfur Clusters. *J Chem Inf Model* **55**, 1508-1508

6. Rappe, A. K., Casewit, C. J., Colwell, K. S., Goddard, W. a., and Skiff, W. M. (1992) UFF, a full periodic table force field for molecular mechanics and molecular dynamics simulations. *Journal of the American Chemical Society* **114**, 10024-10035

7. Baaden, M., Burgard, M., and Wipff, G. (2001) TBP at the Water−Oil Interface:  The Effect of TBP Concentration and Water Acidity Investigated by Molecular Dynamics Simulations. *The Journal of Physical Chemistry B* **105**, 11131-11141

8. Case, D. A., Aktulga, H. M., Belfon, K., Cerutti, D. S., Cisneros, G. A., Cruzeiro, V. W. D., Forouzesh, N., Giese, T. J., Götz, A. W., Gohlke, H., Izadi, S., Kasavajhala, K., Kaymak, M. C., King, E., Kurtzman, T., Lee, T.-S., Li, P., Liu, J., Luchko, T., Luo, R., Manathunga, M., Machado, M. R., Nguyen, H. M., O’Hearn, K. A., Onufriev, A. V., Pan, F., Pantano, S., Qi, R., Rahnamoun, A., Risheh, A., Schott-Verdugo, S., Shajan, A., Swails, J., Wang, J., Wei, H., Wu, X., Wu, Y., Zhang, S., Zhao, S., Zhu, Q., Cheatham, T. E., III, Roe, D. R., Roitberg, A., Simmerling, C., York, D. M., Nagan, M. C., and Merz, K. M., Jr. (2023) AmberTools. *J Chem Inf Model* **63**, 6183-6191

9. Miller, B. R., 3rd, McGee, T. D., Jr., Swails, J. M., Homeyer, N., Gohlke, H., and Roitberg, A. E. (2012) MMPBSA.py: An Efficient Program for End-State Free Energy Calculations. *J Chem Theory Comput* **8**, 3314-3321
